# Decoding Resistin Gene Polymorphisms: Implications for Lung Cancer Risk and Clinical Outcomes of Platinum-Based Chemotherapy

**DOI:** 10.3390/biomedicines13020291

**Published:** 2025-01-24

**Authors:** Weijing Gong, Dandan Huang, Tao Zhou, Xinxin Zhu, Yifei Huang, Yongning Lv, Yu Zhang, Zhaoqian Liu, Fang Zeng, Sanlan Wu

**Affiliations:** 1 Department of Pharmacy, Union Hospital, Tongji Medical College, Huazhong University of Science and Technology, Wuhan 430022, China; weijinggong@hust.edu.cn (W.G.); zbqzht@163.com (T.Z.); yf_huang2012@163.com (Y.H.); luyn_union@163.com (Y.L.); zhangwkp@163.com (Y.Z.); 2 Hubei Province Clinical Research Center for Precision Medicine for Critical Illness, Wuhan 430022, China; 3 Department of Neurosurgery, Union Hospital, Tongji Medical College, Huazhong University of Science and Technology, Wuhan 430022, China; huang960115@126.com; 4 School of Pharmacy, Tongji Medical College, Huazhong University of Science and Technology, Wuhan 430022, China; m202476048@hust.edu.cn; 5 Department of Clinical Pharmacology, Hunan Key Laboratory of Pharmacogenetics, National Clinical Research Center for Geriatric Disorders, Xiangya Hospital, Central South University, Changsha 410017, China; liuzhaoqian63@126.com

**Keywords:** RETN, polymorphism, chemotherapy, prognosis, toxicity

## Abstract

**Background**: Resistin (RETN), an inflammatory cytokine exhibiting multifaceted roles in cancer progression, has emerged as a plausible mediator between inflammation and oncogenesis. Prior research from our group has highlighted the pivotal role of resistin in carcinogenesis and its impact on drug responsiveness. The present study delves into the relationship between resistin expression and genetic polymorphisms with cancer risk and clinical outcomes among lung cancer patients undergoing platinum-based chemotherapy. **Methods**: Immunohistochemical analysis was conducted to assess resistin expression levels in 104 tumor tissues derived from lung adenocarcinoma patients. Additionally, 498 lung cancer patients and 213 healthy controls were recruited for this study, with 467 patients undergoing at least two cycles of platinum-based chemotherapy. Unconditional logistical regression analysis was employed to evaluate the associations between *RETN* polymorphisms and lung cancer risk, as well as clinical outcomes. Genotyping of *RETN* polymorphisms (rs1862513 and rs3745367) was performed using the Sequenom MassARRAY System. **Results**: The findings revealed a positive correlation between resistin expression in tumor tissues and metastasis (particularly distant metastasis) and overall survival in lung adenocarcinoma. However, *RETN* polymorphisms were not significantly associated with overall survival in lung cancer patients. No substantial association was observed between *RETN* polymorphisms and lung cancer risk, chemotherapy response, or toxicities, except for rs1862513, which showed a link with severe gastrointestinal toxicity. Meta-analysis results further confirmed the absence of a significant association between *RETN* polymorphisms and cancer risk. **Conclusions**: Despite the pivotal role of resistin in carcinogenesis, only the *RETN* rs1862513 polymorphism emerges as a potential biomarker for gastrointestinal toxicity in lung cancer patients undergoing platinum-based chemotherapy. However, these findings necessitate validation through well-designed studies with larger sample sizes.

## 1. Introduction

Lung cancer is a leading cause of cancer-related mortality worldwide [1], with a complex etiology involving both genetic and environmental factors. The mechanisms underlying lung cancer development are multifaceted and include genetic mutations, epigenetic alterations, and chronic inflammation [2]. Genetic mutations, such as those in the *EGFR*, *KRAS*, and *ALK* genes, play a crucial role in the initiation and progression of lung cancer by disrupting normal cellular growth and division [3]. Epigenetic changes, including DNA methylation and histone modifications, can further contribute to the development of lung cancer by silencing tumor suppressor genes or activating oncogenes [4]. Chronic inflammation, often resulting from long-term exposure to cigarette smoke or other irritants, can lead to DNA damage and promote the development of cancerous cells [5]. Additionally, the interplay between these mechanisms and the tumor microenvironment further complicates the progression of lung cancer [6]. Furthermore, the late detection of lung cancer, often due to its asymptomatic nature in the early stages, contributes to its high mortality rate. Despite significant advancements in therapeutic strategies, platinum-based chemotherapy remains the cornerstone of treatment for lung cancer patients, particularly those in advanced stages. However, the development of chemoresistance and unpredictable severe side effects pose significant challenges in the management of these patients. Hence, there is an urgent need to identify predictive biomarkers that can differentiate potential beneficiaries of chemotherapy, ensuring maximal efficacy with minimal toxicity [7].

Resistin (RETN), a small secretory molecule, was initially implicated as a potential link between obesity and diabetes. However, extensive molecular research has demonstrated that resistin plays a crucial role in proliferation, metastasis, angiogenesis, inflammation, and metabolic regulation in cancer cells, including those of lung cancer [8]. For instance, resistin enhances the growth and aggressiveness of breast cancer cells by activating the STAT3 pathway [9]. In lung adenocarcinoma, our previous study demonstrated that resistin promotes metastasis via the TLR4/Src/EGFR/PI3K/NF-κB signaling pathway [8]. Additionally, resistin has been shown to enhance angiogenesis in osteosarcoma through the MAPK signaling pathway [10]. Elevated resistin levels have been associated with an increased risk of cancer, particularly obesity-related cancers [11]. Furthermore, resistin has been shown to confer resistance to doxorubicin-induced apoptosis in human breast cancer cells [12], enhance cisplatin-induced cytotoxicity in lung adenocarcinoma cells [13], and contribute to 5-fluorouracil chemoresistance in human colorectal cancer cells [14]. Additionally, resistin may play a role in the development of heart failure and serve as a biomarker for anthracycline-induced cardiotoxicity [15]. These findings suggest that resistin might play a pivotal role in lung cancer development, chemotherapy response, and toxicity.

Single nucleotide polymorphisms (SNPs) can influence gene expression and function, partially explaining individual differences in carcinogenesis, chemotherapy response, and toxicity [16]. Specifically, the *RETN* SNP −420C>G (rs1862513) has been shown to determine monocyte mRNA and serum levels of resistin [17], while the *RETN* +299G>A (rs3745367) polymorphism is significantly associated with resistin levels [18]. To date, only a few studies have focused on the association between *RETN* SNPs and lung cancer susceptibility, as well as clinical outcomes in lung cancer patients undergoing platinum-based chemotherapy.

In the present study, we aimed to investigate the correlation between resistin expression and polymorphisms with overall survival in lung cancer patients. We conducted a hospital-based case–control study to analyze the genotypes of resistin polymorphisms and explored their association with lung cancer risk, efficacy, and toxicity in lung cancer patients receiving platinum-based chemotherapy.

## 2. Materials and Methods

### 2.1. Study Population

In the resistin tumor tissue expression analysis, 104 Chinese Han individuals diagnosed with lung adenocarcinoma were recruited from Xiangya Hospital of Central South University between 2011 and 2012. Each patient underwent histological or cytological confirmation of primary lung adenocarcinoma and staging at the time of surgery, adhering to the guidelines set by the National Comprehensive Cancer Network. These 104 specimens were preserved using routine fixation with 10% formalin and embedded in paraffin. Comprehensive demographic and clinical data, encompassing age, gender, tumor–node–metastasis (TNM) stage, tumor differentiation, smoking history, and comorbidities, were gathered for each patient. Follow-up for these 104 lung adenocarcinoma patients was conducted for at least five years, with telephone check-ins every three months and in-person visits at their residence registration.

For the *RETN* SNPs association study, the research encompassed 711 subjects divided into two distinct groups: a patient group comprising 498 lung cancer patients and a control group consisting of 213 unrelated healthy volunteers. From November 2011 to May 2013, all patients were diagnosed with primary lung cancer through histological or cytological means at either Xiangya Hospital of Central South University or the Affiliated Cancer Hospital of Central South University. Exclusion criteria for the study included pregnancy, lactation, active infection, symptomatic brain or leptomeningeal metastasis, and any previous or concurrent malignancies. Healthy individuals were at least 18 years old and recruited during the same period from the physical examination center of Xiangya Hospital of Central South University [19].

Among the selected lung cancer patients, 467 underwent at least two cycles of platinum-based chemotherapy. These patients did not receive radiotherapy or biological therapy before or during chemotherapy and participated in comprehensive follow-up. Before initiating chemotherapy, patients underwent a comprehensive evaluation to assess their overall health, cancer stage, and potential risks. The selected patients received various platinum-based chemotherapy regimens, including combinations such as platinum + gemcitabine (GP), platinum + etoposide (EP), platinum + docetaxel (DP), platinum + paclitaxel (TP), and platinum + pemetrexed (PP). Additional platinum-based combinations, such as platinum + irinotecan and platinum + vinorelbine, were also utilized. During and after the infusion, patients were closely monitored for any immediate side effects. Chemotherapy was administered in cycles, with a treatment period followed by a rest period of approximately 21 days to allow the body to recover. Following the completion of treatment, patients underwent follow-up assessments to evaluate the effectiveness of the chemotherapy and to monitor for any long-term side effects. Chemotherapy responses were evaluated according to the Response Evaluation Criteria in Solid Tumors (RECIST) guidelines [20]. Patients achieving complete response (CR) or partial response (PR) were considered responders, whereas those with stable disease (SD) or progressive disease (PD) were classified as non-responders. The severity of toxicity was assessed using the National Cancer Institute Common Toxicity Criteria version 3.0 [21]. Platinum-based chemotherapy toxicity primarily involved gastrointestinal and hematological toxicity, with grade 3 or 4 toxicity considered severe. Patients experiencing any severe toxicity were deemed to have suffered from severe overall toxicity. Demographic and clinical information was gathered from medical records and follow-up data. The follow-up period for 227 patients was five years or until death.

The study protocol received approval from the Ethics Committee of Xiangya School of Medicine, Central South University (registration number: CTXY-110008-2) [19].

### 2.2. DNA Extraction, and Genotyping

Genomic DNA was extracted from 5 mL of venous blood utilizing the Genomic DNA Purification Kit (Promega, Madison, WI, USA), adhering strictly to the provided protocol. The extracted genomic DNA samples were then stored at −20 degrees Celsius until required for further use. *RETN* polymorphisms were subsequently genotyped employing the Sequenom MassARRAY System (Sequenom, San Diego, CA, USA). Briefly, the genotyping procedures include the following steps: polymerase chain reaction amplification, shrimp alkaline phosphatase treatment, single base extension, resin purification, MALDI-TOF mass spectrometry, and fragment analysis [22].

### 2.3. Immunohistochemical Staining and Image Analysis

Paraffin-embedded sections of human lung adenocarcinoma tissue were deparaffinized using xylene and subsequently rehydrated through a graded series of ethanol solutions. To neutralize endogenous peroxidase activity, the sections were treated with 3% hydrogen peroxide in methanol. All sections underwent heat-induced antigen retrieval using a sodium citrate buffer solution (0.01 M, pH 6.0). Following thorough washing with phosphate-buffered saline (PBS), the slides were incubated with a human resistin antibody (sc-376336, Santa Cruz Biotechnology, Santa Cruz, CA, USA) at a 1:500 dilution overnight at 4 °C in humidified chambers. The antibody binding was visualized using 3,3′-diaminobenzidine tetrahydrochloride solution (Sigma, St. Louis, MI, USA), followed by counterstaining with hematoxylin.

The immunohistochemical results were independently scored by two experts from the Department of Pathology at Xiangya Hospital, using the H-score method. The scoring was based on both the percentage of positive cells and the intensity of staining [23]. Specifically, positive cell rates of 0–5%, 5–30%, 30–50%, and >50% were assigned scores of 0, 1, 2, 3, and 4, respectively. The staining intensity was categorized as follows: no staining (score 0), faint yellow staining (score 1), moderate buff staining (score 2), and intense brown staining (score 3). The H-score was calculated as the product of the positive cell rate score and the staining intensity score. An H-score of <2 was considered indicative of negative expression, while an H-score of ≥2 was considered positive expression.

### 2.4. Publication Search, Inclusion Criteria, and Data Extraction

A comprehensive literature review was conducted using PubMed (https://pubmed.ncbi.nlm.nih.gov/), Embase (https://www.embase.com/), and Google Scholar (https://scholar.google.com/) up to 20 March 2024. The search was tailored using the following combined terms: “resistin or RETN” AND “polymorphism or SNP or variant” AND “cancer or tumor or neoplasm or carcinoma”.

The inclusion criteria for the studies were as follows: (1) original case–control studies examining the association between *RETN* polymorphisms and cancer; (2) studies that provided detailed genotype frequencies for both cases and controls, or data sufficient to calculate these frequencies. Studies lacking sufficient data or those where the control group deviated from the Hardy–Weinberg equilibrium were excluded. Two independent researchers extracted and evaluated the data using the Newcastle–Ottawa scale (NOS).

### 2.5. Statistical Analysis

In our association study, categorical variables were analyzed using the chi-square and Student’s *t*-test to assess differences in proportions between groups. The Hardy–Weinberg equilibrium was calculated for the control group. To estimate the association between *RETN* SNPs and lung cancer susceptibility, chemotherapy response, and toxicity, unconditional logistic regression with adjustments was employed, yielding odds ratios (ORs) and 95% confidence intervals (CIs). The statistical analyses were performed using PLINK 1.9 and R version 4.0.5. Kaplan–Meier curves were utilized to generate survival plots, and Cox’s proportional hazards model was applied for both univariate and multivariate survival analyses. Data analysis was facilitated by PASW Statistics v18.0 (IBM Co., Armonk, NY, USA). All tests were conducted on a two-sided basis, with statistical significance set at *p* < 0.05.

For the meta-analysis, the risk of cancer associated with *RETN* SNPs was estimated by calculating pooled ORs and 95% CIs. Heterogeneity in effect size among studies was assessed using Cochrane’s Q test and I^2^ test. A fixed effect model was selected if I^2^ < 50% and *p* > 0.10; otherwise, a random effect model was utilized. Publication bias was evaluated through funnel plots and Egger’s test, with statistical significance set at *p* < 0.05. These statistical analyses were performed using R version 4.0.5.

## 3. Results

### 3.1. Association of RETN SNPs with Lung Cancer Susceptibility

The flow chart of this study is presented in Supplemental Figure S1. The characteristics of lung cancer patients and healthy controls are presented in Supplemental Table S1. Among the 498 cases, 189 (37.9%) patients were diagnosed with squamous cell carcinoma (SCC), 217 (43.6%) with adenocarcinoma (ADC), and 69 (13.9%) with small cell lung cancer (SCLC). Statistically significant differences were observed in the age and gender distributions between cases and controls. The genotype distribution of *RETN* SNPs in healthy controls adhered to the Hardy–Weinberg Equilibrium (HWE) for rs1862513 (*p* = 0.225) and rs3745367 (*p* = 0.632).

**Figure 1:**
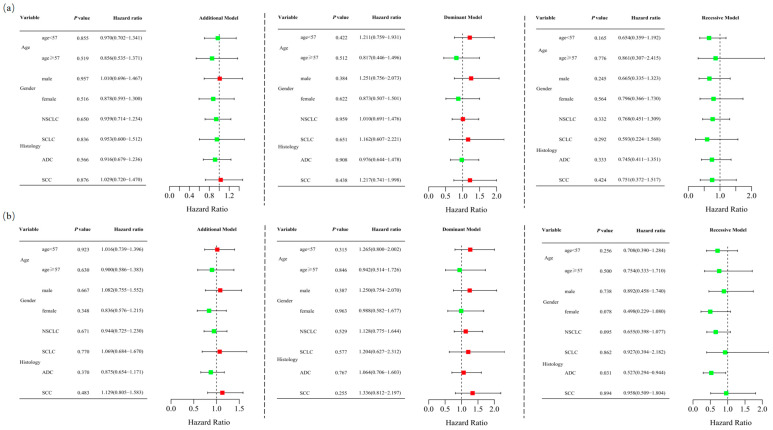
Stratified analyses were conducted to assess the association between *RETN* rs1862513 (**a**) and rs3745367 (**b**) and lung cancer risk using additive, dominant, and recessive models, with adjustments for age and sex. Each box and its horizontal line depict the odds ratio (OR) and the 95% confidence interval (CI). The green color signifies hazard ratios below 1, whereas the red color indicates hazard ratios above 1. Abbreviations include NSCLC for non-small cell lung carcinoma, ADC for adenocarcinoma, SCC for squamous cell carcinoma, and SCLC for small cell lung cancer.

Unconditional logistic regression analysis, adjusted for age and sex, revealed no significant association between *RETN* SNPs and lung cancer risk in the additive (rs1862513: adjusted OR = 0.935, 95% CI = 0.716–1.221, *p* = 0.623; rs3745367: adjusted OR = 0.977, 95% CI = 0.757–1.261, *p* = 0.857), dominant (rs1862513: adjusted OR = 1.032, 95% CI = 0.714–1.491, *p* = 0.868; rs3745367: adjusted OR = 1.127, 95% CI = 0.783–1.621, *p* = 0.521), and recessive (rs1862513: adjusted OR = 0.726, 95% CI = 0.433–1.218, *p* = 0.225; rs3745367: adjusted OR = 0.744, 95% CI = 0.460–1.202, *p* = 0.227) models (Table 1). Stratified analysis showed that rs3745367 was significantly associated with ADC risk in the recessive model (adjusted OR = 0.527, 95% CI = 0.294–0.944, *p* = 0.031) (Figure 1).

A meta-analysis was conducted to investigate the association between *RETN* SNPs and cancer risk. Thirteen studies met all inclusion criteria and were included: thirteen studies for rs1862513, comprising 4068 cases and 4388 controls, and six studies for rs3745367, encompassing 2543 cases and 2652 controls [24,25,26,27,28,29,30,31,32,33,34,35,36]. The characteristics of the included studies are detailed in Supplementary Table S2. The summary of all calculated results is presented in Table 2. No significant association was found between *RETN* rs1862513 and cancer risk in codominant, dominant, recessive, overdominant, and allelic models. However, rs3745367 was associated with cancer susceptibility only in the codominant model (GA vs. GG, OR = 1.183, 95% CI = 1.013–1.382, *p* = 0.034), although publication bias might be present (Egger’s test *p* = 0.012, Begg’s test *p* = 0.060) (Table 2, Supplementary Figures S2 and S3).

### 3.2. Association of RETN SNPs with Platinum-Based Chemotherapy Response in Lung Cancer Patients

**Figure 2:**
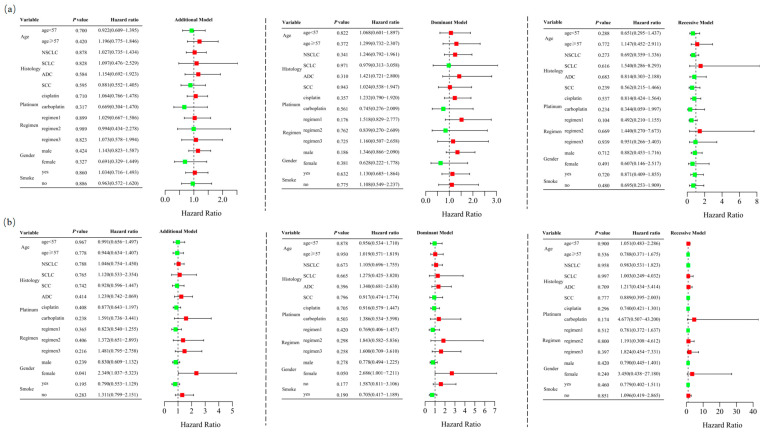
Stratified analyses were performed to evaluate association of *RETN* rs1862513 (**a**) and rs3745367 (**b**) with response to platinum-based chemotherapy in lung cancer patients. Analyses were conducted using additive, dominant, and recessive models, with adjustments for age, sex, stage, histological type, smoking status, and chemotherapy regimens. Each box and its horizontal line represent the odds ratio (OR) and the 95% confidence interval (CI). The green color signifies hazard ratios below 1, whereas the red color indicates hazard ratios above 1. Abbreviations include NSCLC for non-small cell lung carcinoma, ADC for adenocarcinoma, SCC for squamous cell carcinoma, and SCLC for small cell lung cancer. Chemotherapy regimens are as follows: Regimen 1, platinum + gemcitabine; Regimen 2, platinum + etoposide; Regimen 3, platinum + pemetrexed.

Among the 467 patients who underwent at least two cycles of platinum-based chemotherapy, 184 (39.4%) responded positively, while 283 (60.6%) exhibited a poor response. The characteristics of these 467 cases are detailed in Supplementary Table S3. Unconditional logistic regression analysis, adjusted for age, sex, stage, histological type, smoking status, and chemotherapy regimens, indicated that *RETN* SNPs were not significantly associated with the short-term response to platinum-based chemotherapy in lung cancer patients in the additive (rs1862513: adjusted OR = 1.022, 95% CI = 0.760–1.374, *p* = 0.884; rs3745367: adjusted OR = 0.965, 95% CI = 0.728–1.279, *p* = 0.803), dominant (rs1862513: adjusted OR = 1.170, 95% CI = 0.786–1.743, *p* = 0.438; rs3745367: adjusted OR = 0.998, 95% CI = 0.667–1.494, *p* = 0.992), and recessive (rs1862513: adjusted OR = 0.768, 95% CI = 0.424–1.391, *p* = 0.383; rs3745367: adjusted OR = 0.882, 95% CI = 0.518–1.503, *p* = 0.645) models (Table 1). Stratified analysis revealed a significant association between rs3745367 and the chemotherapy response in female patients in the additive model (adjusted OR = 2.349, 95% CI = 1.037–5.323, *p* = 0.041) (Figure 2).

### 3.3. Association of RETN SNPs with Platinum-Based Chemotherapy Toxicity in Lung Cancer Patients

Among the 467 patients who underwent at least two cycles of platinum-based chemotherapy, 101 (21.6%) and 114 (24.4%) experienced severe gastrointestinal and hematological toxicities, respectively, according to RECIST criteria. A total of 181 (38.8%) patients suffered from at least one type of severe toxicity.

**Figure 3:**
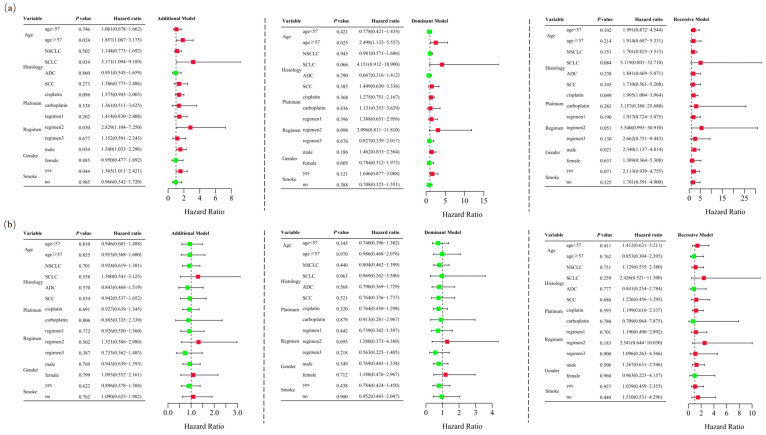
Stratified analyses were conducted to examine association between *RETN* rs1862513 (**a**) and rs3745367 (**b**) and gastrointestinal toxicity in lung cancer patients receiving platinum-based chemotherapy. Analyses utilized additive, dominant, and recessive models, with adjustments for age, sex, stage, histological type, smoking status, and chemotherapy regimens. Each box and its horizontal line indicate the odds ratio (OR) and the 95% confidence interval (CI). The green color signifies hazard ratios below 1, whereas the red color indicates hazard ratios above 1. Abbreviations include NSCLC for non-small cell lung carcinoma, ADC for adenocarcinoma, SCC for squamous cell carcinoma, and SCLC for small cell lung cancer. Chemotherapy regimens are as follows: Regimen 1, platinum + gemcitabine; Regimen 2, platinum + etoposide; Regimen 3, platinum + pemetrexed.

Unconditional logistic regression analysis, adjusted for age, sex, stage, histological type, smoking status, and chemotherapy regimens, revealed that *RETN* SNPs were not significantly associated with severe gastrointestinal toxicity in lung cancer patients undergoing platinum-based chemotherapy in the additive (rs1862513: adjusted OR = 1.332, 95% CI = 0.945–1.878, *p* = 0.102; rs3745367: adjusted OR = 0.956, 95% CI = 0.680–1.343, *p* = 0.796), dominant (rs1862513: adjusted OR = 1.226, 95% CI = 0.761–1.975, *p* = 0.402; rs3745367: adjusted OR = 0.838, 95% CI = 0.521–1.347, *p* = 0.465), and recessive (rs3745367: adjusted OR = 1.173, 95% CI = 0.623–2.207, *p* = 0.621) models. However, rs1862513 (note: corrected from rs1852513 as per context) was found to be associated with severe gastrointestinal toxicity in the recessive model (adjusted OR = 1.948, 95% CI = 1.030–3.687, *p* = 0.040) (Table 1). Subsequent stratified analysis showed that rs1862513 was associated with an increased risk of severe gastrointestinal toxicity in patients aged 57 or older, those with SCLC, those receiving cisplatin, those on a platinum + etoposide regimen, males, and smokers (Figure 3).

**Figure 4:**
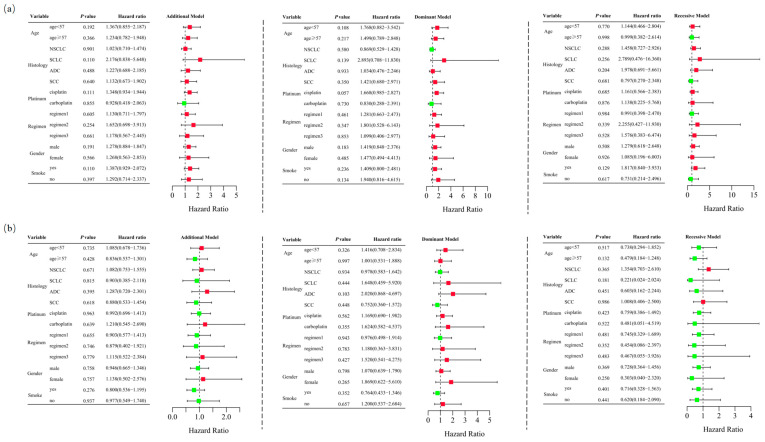
Stratified analyses were performed to assess association between *RETN* rs1862513 (**a**) and rs3745367 (**b**) and hematological toxicity in lung cancer patients undergoing platinum-based chemotherapy. Analyses were conducted using additive, dominant, and recessive models, with adjustments for age, sex, stage, histological type, smoking status, and chemotherapy regimens. Each box and its horizontal line indicate the odds ratio (OR) and the 95% confidence interval (CI). The green color signifies hazard ratios below 1, whereas the red color indicates hazard ratios above 1. Abbreviations include NSCLC for non-small cell lung carcinoma, ADC for adenocarcinoma, SCC for squamous cell carcinoma, and SCLC for small cell lung cancer. Chemotherapy regimens are as follows: Regimen 1, platinum + gemcitabine; Regimen 2, platinum + etoposide; Regimen 3, platinum + pemetrexed.

Logistic regression analysis demonstrated that *RETN* SNPs were not significantly associated with severe hematological toxicity induced by platinum-based chemotherapy in the additive (rs1862513: adjusted OR = 1.260, 95% CI = 0.909–1.747, *p* = 0.165; rs3745367: adjusted OR = 0.987, 95% CI = 0.719–1.355, *p* = 0.935), dominant (rs1862513: adjusted OR = 1.434, 95% CI = 0.907–2.267, *p* = 0.123; rs3745367: adjusted OR = 1.193, 95% CI = 0.753–1.888, *p* = 0.453), and recessive (rs1862513: adjusted OR = 1.188, 95% CI = 0.620–2.274, *p* = 0.604; rs3745367: adjusted OR = 0.688, 95% CI = 0.363–1.307, *p* = 0.254) models (Table 1). Stratified analysis also failed to show an association between *RETN* SNPs and severe hematological toxicity (Figure 4).

After adjusting for age, sex, stage, histological type, smoking status, and chemotherapy regimens, *RETN* SNPs were not significantly associated with overall toxicity in the additive (rs1862513: adjusted OR = 1.295, 95% CI = 0.965–1.737, *p* = 0.085; rs3745367: adjusted OR = 0.899, 95% CI = 0.677–1.194, *p* = 0.461), dominant (rs1862513: adjusted OR = 1.451, 95% CI = 0.971–2.169, *p* = 0.069; rs3745367: adjusted OR = 0.930, 95% CI = 0.622–1.390, *p* = 0.723), and recessive (rs1862513: adjusted OR = 1.266, 95% CI = 0.702–2.282, *p* = 0.432; rs3745367: adjusted OR = 0.770, 95% CI = 0.444–1.336, *p* = 0.352) models (Table 1). Stratified analysis further confirmed that *RETN* SNPs were not associated with overall toxicity in lung cancer patients undergoing platinum-based chemotherapy (Supplemental Figure S4).

### 3.4. Association of Resistin Expression and SNPs with Overall Survival in Lung Cancer Patients

Immunohistochemical analysis was conducted to detect the expression of resistin in 104 lung adenocarcinoma tissues (Supplemental Figure S5a). Results revealed that 65.38% of these tissues exhibited positive resistin expression, with an H-score of ≥2. The study further investigated the correlation between resistin expression and various clinicopathological variables, including age, sex, smoking status, comorbid conditions, clinical stage, differentiation, lymph node metastasis, and total metastasis. Notably, a significantly positive correlation was observed between resistin expression in lung adenocarcinoma tissues and both other metastasis (excluding lymph node metastasis) (*p* < 0.01) and overall metastasis (*p* < 0.01) (Table 3).

The Kaplan–Meier method was employed to explore the relationship between resistin expression in lung adenocarcinoma tissues and overall survival. Patients with negative resistin expression exhibited significantly higher overall survival, with a median survival of 35.07 months compared to 17.60 months for those with positive resistin expression (*p* < 0.01) (Supplemental Figure S5b).

To assess the factors influencing resistin expression, univariate and multivariable Cox regression analyses were conducted to estimate hazard ratios (HRs) and 95% confidence intervals (CIs). In the univariate analysis, significant factors associated with overall survival included resistin expression (HR = 2.343, 95% CI = 1.460–3.759, *p* < 0.01), metastasis (HR = 1.986, 95% CI = 1.258–3.136, *p* < 0.01), and stage (HR = 1.620, 95% CI = 1.008–2.604, *p* < 0.05). However, after adjusting for other variables in the multivariable analysis, only resistin expression emerged as an independent predictor of overall survival in lung adenocarcinoma patients (HR = 1.922, 95% CI = 1.008–3.393, *p* < 0.05) (Table 4).

In a separate study on *RETN* polymorphisms, 227 lung cancer patients who underwent at least two cycles of chemotherapy were included. However, no significant association was found between *RETN* polymorphisms and overall survival in these lung cancer patients (Supplemental Figure S5c,d).

## 4. Discussion

Resistin, a pro-inflammatory cytokine, plays a multifaceted role in cancer development and treatment. In our study, we delved into the potential impact of *RETN* polymorphisms on lung cancer risk and chemotherapy outcomes. Our findings revealed that *RETN* SNPs were not significantly associated with cancer susceptibility, chemotherapy response, or severe toxicities in lung cancer patients, with the exception of rs1862513, which showed a link to severe gastrointestinal toxicity in a recessive model. Conversely, the expression of resistin in tumor tissue was found to be correlated with metastasis, particularly distant metastasis, and overall survival in lung adenocarcinoma. However, *RETN* SNPs did not exhibit a significant association with overall survival in lung cancer patients.

Proinflammatory cytokines, such as tumor necrosis factor-alpha (TNF-α), interleukin-1 beta (IL-1β), and interleukin-6 (IL-6), play a crucial role in shaping a proinflammatory tumor microenvironment. This particular milieu provides favorable conditions that fuel tumor growth, invasion, and the spread of metastases. These cytokines act as triggers, activating the NF-κB and STAT3 inflammatory pathways. Through a series of complex mechanisms including immune evasion, tumor angiogenesis, epithelial–mesenchymal transition, and modulation of apoptosis, these pathways drive the initiation, progression, and metastatic spread of lung cancer [37]. Resistin, another important proinflammatory factor, has been shown to play a vital role in carcinogenesis, promoting tumor growth and metastasis by modulating cellular phenotypes and the tumor microenvironment [8,38]. Both rs1862513 and rs3745367 have been shown to be associated with resistin levels [17,18]. The rs1862513 variant has been reported to be associated with an increased risk of colorectal cancer in Iran [27] and Czech Republic [32]. However, this association was not observed in studies conducted in Sweden [24], Turkey [35], or Serbia [39]. Rs1862513 has been shown to be associated with the risk of breast cancer in both China [33] and Iran [36]. It was associated with the risk of endometrial cancer in Turkey [31], but not oral squamous cell carcinoma in China [34]. The rs3745367 variant was associated with the risk of colon cancer in Saudi Arabia [25], but not colorectal cancer in Iran [28] or breast cancer in China [33]. We investigated the role of *RETN* rs1862513 and rs3745367 in lung cancer. Surprisingly, our results indicated that these SNPs were not associated with lung cancer susceptibility. Previous studies examining the relationship between *RETN* polymorphisms and cancer risk have yielded inconsistent findings. To clarify this association, we conducted a meta-analysis, which revealed that neither rs1862513 nor rs3745367 were significantly linked to cancer risk, except for rs3745367 in a codominant model. Our results were inconsistent with the previous meta-analysis of nine studies including 1951 cases and 2295 controls [40]. Our meta-analysis contained larger samples and recent studies. However, this finding should be interpreted cautiously due to potential publication bias and lack of adjustments.

Despite the potential risks, platinum-based drugs remain a cornerstone in lung cancer treatment. Firstly, they are highly effective against a broad range of lung cancer types. The broad-spectrum activity of platinum-based drugs makes them a standard first-line treatment for patients with advanced NSCLC who have a good performance status. Secondly, platinum-based chemotherapy has been shown to improve overall survival and progression-free survival in patients with lung cancer. This is particularly important in cases where targeted therapies, such as EGFR-TKIs, may not be effective or available. Additionally, platinum-based chemotherapy can be effectively combined with other treatment modalities, such as immunotherapy and targeted therapies, to enhance treatment outcomes. While platinum-based chemotherapy does have side effects, its established efficacy and the ability to be tailored to individual patient needs make it a valuable component of lung cancer treatment regimens. Previous studies have explored the impact of resistin on cytotoxicity induced by chemotherapy drugs such as doxorubicin, cisplatin, and 5-Fluorouracil. Nevertheless, there is limited research on the association between *RETN* SNPs and chemotherapy response and toxicity. In our study, we assessed the role of *RETN* SNPs in platinum-based chemotherapy outcomes in lung cancer patients. Our results showed no significant associations, except for rs1862513, which was linked to severe gastrointestinal toxicity. It is noteworthy that an upregulation of resistin expression in gastrointestinal tract cells has been observed in gastrointestinal disorders, suggesting a potential link between obesity and cancer [41]. Furthermore, the rs1862513 polymorphism has been positively correlated with resistin expression [42].

Previous studies have investigated serum or plasma resistin levels in various cancers, finding a positive association with cancer risk. However, there is less research on resistin expression in tumor tissues. In our study, we explored resistin expression in lung adenocarcinoma and found a positive correlation with metastasis, particularly distant metastasis, and overall survival. These findings align with previous research [43]. Our previous study also demonstrated that resistin promotes lung adenocarcinoma metastasis through the TLR4/Src/EGFR/PI3K/NF-κB pathway [8]. Additionally, resistin has been negatively correlated with overall survival in several studies [43,44,45,46]. However, our exploration of the association between *RETN* SNPs and overall survival yielded no significant findings. This may be attributed to other factors that significantly influence resistin expression beyond polymorphisms. Drugs such as dexamethasone could significantly alter the protein and mRNA levels of resistin irrespective of *RETN* polymorphisms [47]. Environmental Factors such as Bisphenol A have been proved to affect the expression of resistin [48]. Animal studies have shown that lifestyle elements such as chronic alcohol consumption and a high-fat diet could also affect resistin expression levels [49,50].

## 5. Conclusions

In our study, we investigated the impact of *RETN* SNPs on the progression of lung cancer and the clinical outcomes of lung cancer patients undergoing platinum-based chemotherapy. Despite conducting thorough analyses, no significant associations were identified, except for rs1862513, which was linked to severe gastrointestinal toxicity. Additionally, we found that the expression of resistin in tumor tissues correlated with metastasis (particularly distant metastasis) and overall survival in patients with lung adenocarcinoma. However, *RETN* SNPs were not associated with overall survival in lung cancer patients. Therefore, it is imperative to validate these findings in future, well-designed studies with larger sample sizes.

## Figures and Tables

**Table 1:** Association of *RETN* SNPs with lung cancer susceptibility and clinical outcomes in lung cancer patients with platinum-based chemotherapy.

Type	Genotype	n (%)	n (%)	Additive Model		Dominant Model		Recessive Model	
				OR (95% CI)	*p*	OR (95% CI)	*p*	OR (95% CI)	*p*
Risk		Case	Control						
	rs1862513			0.935 (0.716–1.221)	0.623	1.032 (0.714–1.491)	0.868	0.726 (0.433–1.218)	0.225
	GG	56 (11.2)	33 (15.5)						
	GC	243 (48.8)	98 (46.0)						
	CC	188 (37.8)	78 (36.6)						
	rs3745367			0.977 (0.757–1.261)	0.857	1.127 (0.783–1.621)	0.521	0.744 (0.460–1.202)	0.227
	AA	71 (14.3)	37 (17.4)						
	GA	239 (48.0)	86 (40.4)						
	GG	180 (36.1)	85 (39.9)						
Chemotherapy response		Responder	Non-responder						
	rs1862513			1.022 (0.760–1.374)	0.884	1.170 (0.786–1.743)	0.438	0.768 (0.424–1.391)	0.383
	GG	24 (13.0)	29 (10.2)						
	GC	81 (44.0)	143 (50.5)						
	CC	74 (40.2)	105 (37.1)						
	rs3745367			0.965 (0.728–1.279)	0.803	0.998 (0.667–1.494)	0.992	0.882 (0.518–1.503)	0.645
	AA	30 (16.3)	39 (13.8)						
	GA	87 (47.3)	135 (47.7)						
	GG	64 (34.8)	105 (37.1)						
Gastrointestinal toxicity		Grade 0–2	Grade 3–4						
	rs1862513			1.332 (0.945–1.878)	0.102	1.226 (0.761–1.975)	0.402	1.948 (1.030–3.687)	0.04
	GG	35 (9.6)	19 (18.8)						
	GC	178 (48.6)	47 (46.5)						
	CC	142 (38.8)	35 (34.7)						
	rs3745367			0.956 (0.680–1.343)	0.796	0.838 (0.521–1.347)	0.465	1.173 (0.623–2.207)	0.621
	AA	53 (14.5)	16 (15.8)						
	GA	177 (48.4)	45 (44.6)						
	GG	131 (35.8)	38 (37.6)						
Hematological toxicity		Grade 0–2	Grade 3–4						
	rs1862513			1.260 (0.909–1.747)	0.165	1.434 (0.907–2.267)	0.123	1.188 (0.620–2.274)	0.604
	GG	38 (10.8)	16 (14.0)						
	GC	164 (46.5)	61 (53.5)						
	CC	142 (40.2)	35 (30.7)						
	rs3745367			0.987 (0.719–1.355)	0.935	1.193 (0.753–1.888)	0.453	0.688 (0.363–1.307)	0.254
	AA	55 (15.6)	14 (12.3)						
	GA	161 (45.6)	61 (53.5)						
	GG	131 (37.1)	38 (33.3)						
Overall toxicity		Grade 0–2	Grade 3–4						
	rs1862513			1.295 (0.965–1.737)	0.085	1.451 (0.971–2.169)	0.069	1.266 (0.702–2.282)	0.432
	GG	29 (10.1)	25 (13.8)						
	GC	130 (45.4)	95 (52.5)						
	CC	118 (41.3)	59 (32.6)						
	rs3745367			0.899 (0.677–1.194)	0.461	0.930 (0.622–1.390)	0.723	0.770 (0.444–1.336)	0.352
	AA	45 (15.7)	24 (13.3)						
	GA	134 (46.9)	88 (48.6)						
	GG	102 (35.7)	67 (37.0)						

Abbreviations: n, number; OR, odds ratio; CI, confidence interval.

**Table 2:** Meta-analysis of selected studies evaluating association of *RETN* polymorphisms with cancer susceptibility.

Genetic Model	Test of Association			Test of Heterogeneity			Publication Bias (*p* Value)	
	OR	95% CI	*p* Value	Model	*p* Value	I^2^ (%)	Egger’s Test	Begg’s Test
rs1862513								
Codominant model 1 (GG vs. CC)	1.195	0.958–1.492	0.115	Random	0.020	50	0.218	0.161
Codominant model 2 (CG vs. CC)	1.073	0.976–1.180	0.133	Fixed	0.131	50	0.055	0.058
Codominant model 3 (GG vs. CG)	1.058	0.880–1.273	0.547	Random	0.079	38	0.872	0.951
Dominant model (GG + CG vs. CC)	1.068	0.977–1.167	0.149	Fixed	0.120	24	0.054	0.010
Recessive model (GG vs. CG + CC)	1.100	0.914–1.326	0.313	Random	0.043	44	0.508	0.951
Overdominant model (CG vs. GG + CC)	1.029	0.943–1.124	0.522	Fixed	0.329	12	0.075	0.059
Allelic model (G vs. C)	1.083	0.985–1.190	0.098	Random	0.054	42	0.138	0.246
rs3745367								
Codominant model 1 (AA vs. GG)	1.460	0.753–2.831	0.263	Random	0.001	86	0.264	0.260
Codominant model 2 (GA vs. GG)	1.183	1.013–1.382	0.034	Random	0.029	60	0.012	0.060
Codominant model 3 (AA vs. GA)	1.019	0.658–1.578	0.932	Random	0.001	75	0.770	1.000
Dominant model (AA + GA vs. GG)	1.390	0.944–2.049	0.096	Random	0.001	78	0.052	0.133
Recessive model (AA vs. GA + GG)	1.135	0.688–1.872	0.621	Random	0.001	83	0.630	0.452
Overdominant model (GA vs. GG + AA)	1.109	0.992–1.239	0.069	Fixed	0.125	42	0.244	0.452
Allelic model (A vs. G)	1.215	0.900–1.640	0.204	Random	0.001	86	0.255	0.260

Abbreviations: OR, odds ratio; CI, confidence interval.

**Table 3:** Correlation of resistin expression with clinicopathological characteristics of lung adenocarcinoma patients.

Variables		Number	Negative	Positive	*p* Value
Total		104	36	68	
Age	<60	64	22	42	0.883
	≥60	40	14	26	
Gender	male	52	19	33	0.837
	female	52	17	35	
History of Smoking	no	68	20	48	0.188
	yes	36	16	20	
Complications	no	71	25	46	0.973
	yes	33	11	22	
Stage	I, II	36	16	20	0.076
	III, IV	63	17	46	
Differentiation	low	23	9	14	
	middle	56	17	39	0.614
	high	25	10	15	
Lymph Node Metastasis	no	71	25	46	0.950
	yes	31	10	21	
Other metastasis ^a^	no	69	32	37	0.000 *
	yes	33	3	30	
Metastasis	no	41	22	19	0.002 *
	yes	61	13	48	

Abbreviations: Other metastasis ^a^ = metastasis except lymph node metastasis. * *p* < 0.05.

**Table 4:** Univariate and multivariable analysis of overall survival for lung adenocarcinoma.

Variable	Item	Univariate			Multivariate		
		HR	95%CI	*p*	HR	95%CI	*p*
Resistin	positive	2.343	(1.460, 3.759)	0.000 *	1.922	(1.088, 3.393)	0.024 *
	negative	1.000					
Metastasis	yes	1.986	(1.258, 3.136)	0.003 *	1.166	(0.414, 3.289)	0.771
	no	1.000					
Stage	III, IV	1.620	(1.008, 2.604)	0.046 *	1.103	(0.445, 2.730)	0.833
	I, II	1.000					

Abbreviations: HR = hazard ratios; CI = confidence intervals. * *p* < 0.05.

## Data Availability

The datasets used and/or analyzed during the current study are available from the corresponding author on reasonable request.

## Supplementary Materials

The following supporting information can be downloaded at: https://www.mdpi.com/article/10.3390/biomedicines13020291/s1. Figure S1. Flow chart of this study. Figure S2. Forest plot of the risk of cancer associated with *RETN* rs1862513 polymorphism under codominant model 1 (GG vs. CC) (a), codominant model 2 (CG vs. CC) (b), codominant model 3 (GG vs. CG) (c), dominant model (GG+CG vs. CC) model (d), recessive model (GG vs. CG+CC) (e), overdominant model (CG vs. GG+CC) (f), and allelic model (G vs. C) (g). Figure S3. Forest plot of the risk of cancer associated with *RETN* rs3745367 polymorphism under codominant model 1 (AA vs. GG) (a), codominant model 2 (GA vs. GG) (b), codominant model 3 (AA vs. GA) (c), dominant model (AA+GA vs. GG) model (d), recessive model (AA vs. GA+GG) (e), overdominant model (GA vs. GG+AA) (f), and allelic model (A vs. G) (g). Figure S4. Stratification analyses were conducted to examine the relationship between *RETN* polymorphisms rs1862513 (a) and rs3745367 (b) and overall toxicity in lung cancer patients undergoing platinum-based chemotherapy. Analyses were performed using additive, dominant, and recessive genetic models, while adjusting for various confounding factors including age, sex, disease stage, histological type, smoking status, and specific chemotherapy regimens. Results are depicted with each box and horizontal line representing the odds ratio (OR) and its 95% confidence interval (CI), respectively. The green color signifies hazard ratios below 1, whereas the red color indicates hazard ratios above 1. The study focused on different types of lung cancer, such as non-small cell lung carcinoma (NSCLC), adenocarcinoma (ADC), squamous cell carcinoma (SCC), and small cell lung cancer (SCLC). Chemotherapy regimens considered were: Regimen 1, which combines platinum with gemcitabine; Regimen 2, platinum with etoposide; and Regimen 3, platinum with pemetrexed. Figure S5. Correlation between resistin expression and polymorphism and 5-year overall survival. (a) Representative immunohistochemical staining of resistin expression in human lung adenocarcinoma tissues; (b) overall survival analyses for lung adenocarcinoma patients based on expression of resistin in adenocarcinoma tissues according to Kaplan–Meier method; genotype of *RETN* rs1862513 (c) and rs3745367 (d) and their association with overall survival. Table S1. Clinical characteristics of lung cancer patients and healthy controls. Table S2. Characteristics of included studies on RETN polymorphisms and cancer susceptibility. Table S3. Clinical character of lung cancer patients with platinum-based chemotherapy.
