# Supplementary material for: Decoding Resistin Gene Polymorphisms: Implications for Lung Cancer Risk and Clinical Outcomes of Platinum-Based Chemotherapy
Source: Biomedicines. 2025 Jan 24;13(2):291. doi: 10.3390/biomedicines13020291 (PMC11852191; doi:10.3390/biomedicines13020291)
Supplement: Supplementary file 1 [file biomedicines-13-00291-s001.zip › biomedicines-3385178-supplementary.pdf]

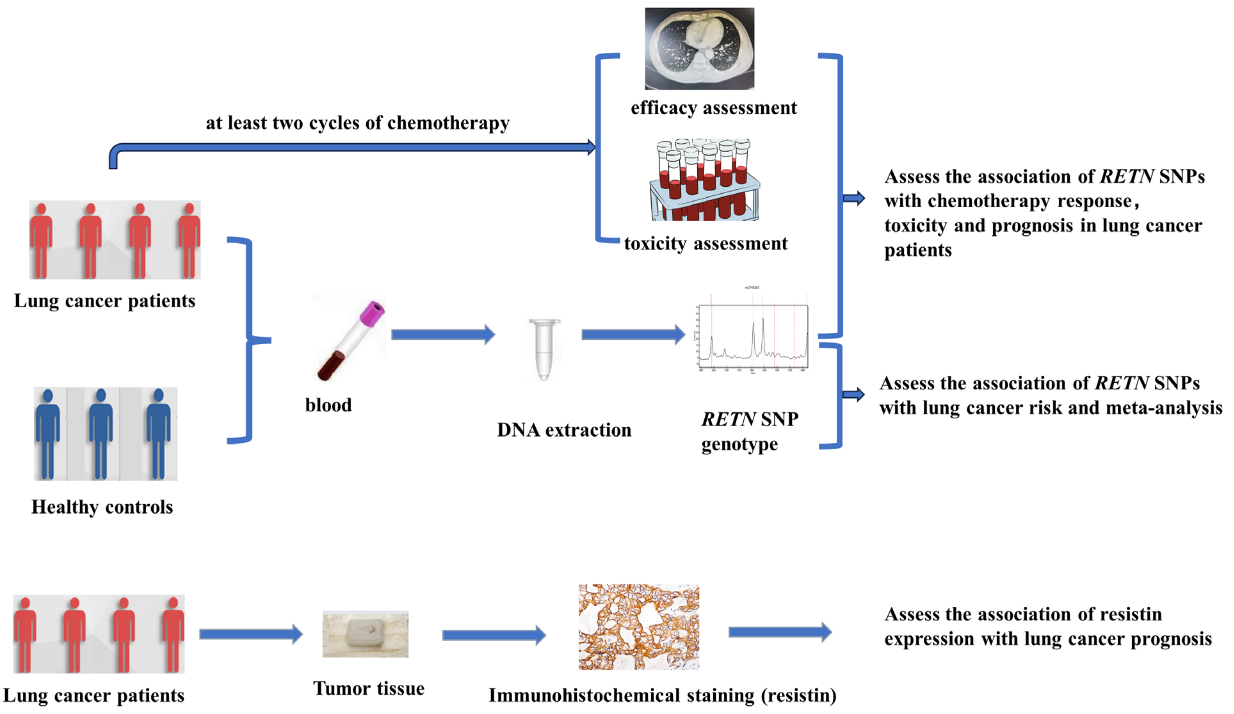

Supplemental Figure S1. The flow chart of this study.

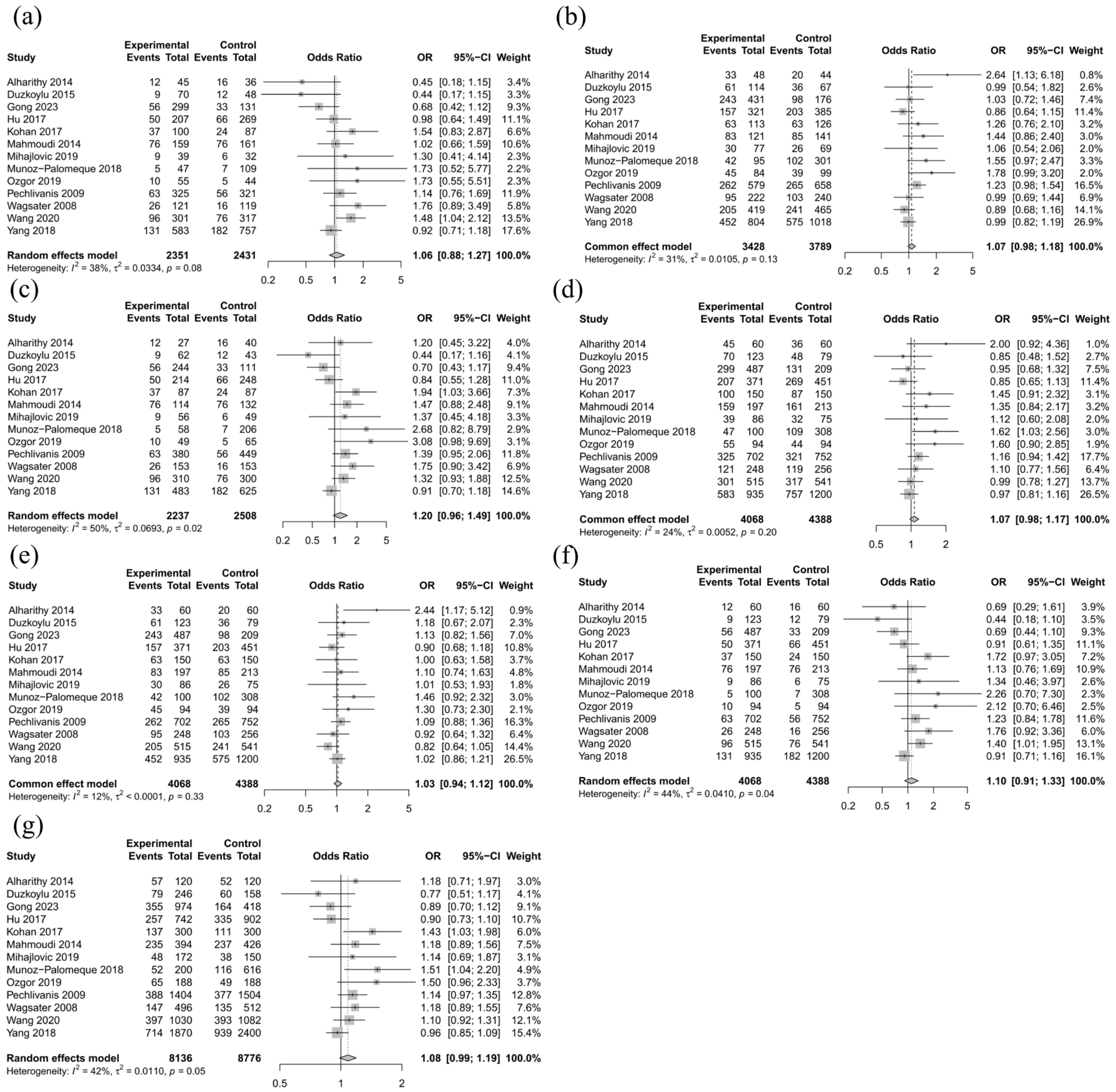

Supplemental Figure S2. Forest plot of the risk of cancer associated with *RETN* rs1862513 polymorphism under codominant model 1 (GG vs. CC) (a), Codominant model 2 (CG vs. CC) (b), Codominant model 3 (GG vs. CG) (c), Dominant model (GG+CG vs. CC) model (d), Recesive model (GG vs. CG+CC) (e), Overdominant model (CG vs. GG+CC) (f), and Allelic model (G vs. C) (g).

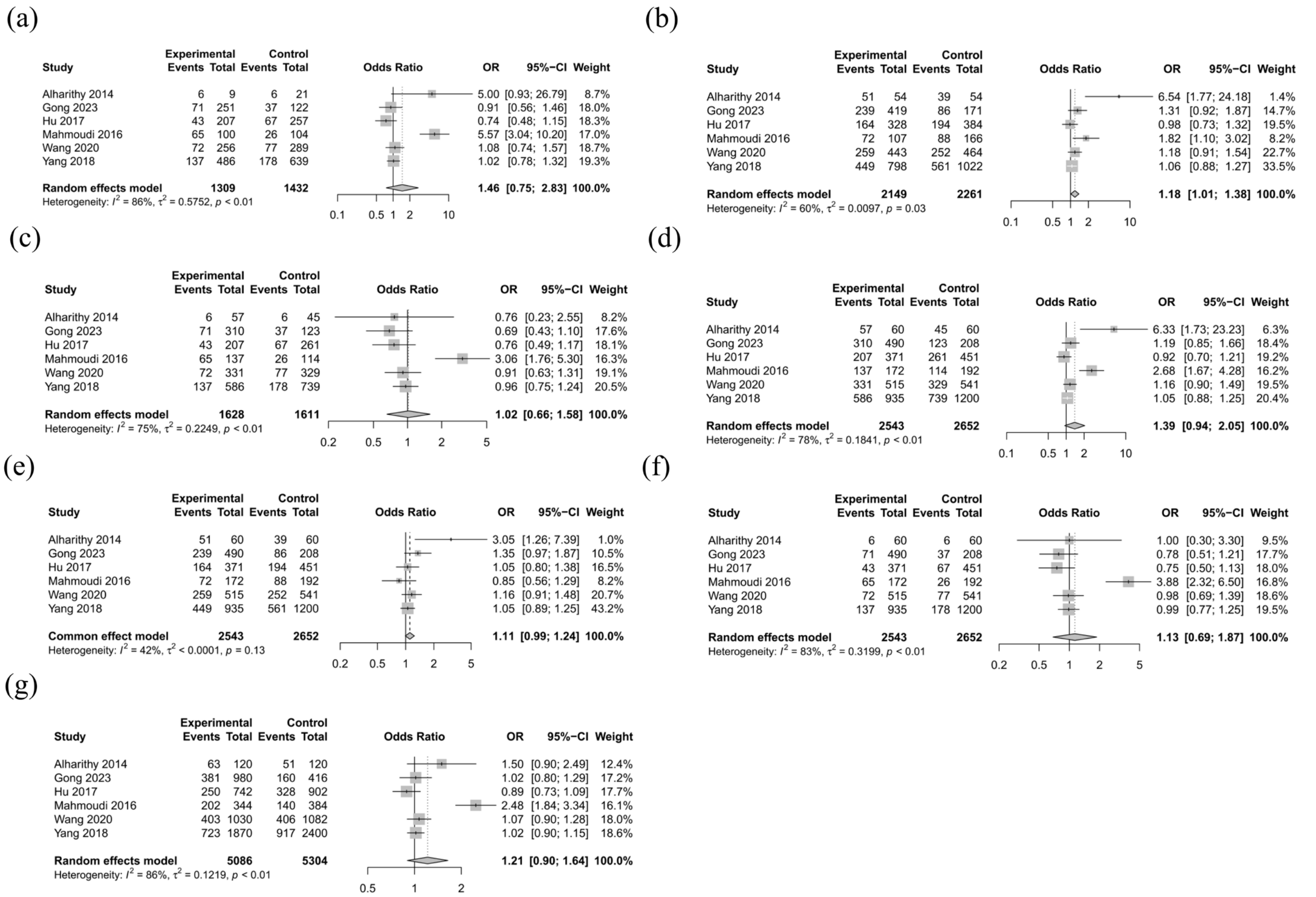

Supplemental Figure S3. Forest plot of the risk of cancer associated with *RETN* rs3745367 polymorphism under codominant model 1 (AA vs. GG) (a), Codominant model 2 (GA vs. GG) (b), Codominant model 3 (AA vs. GA) (c), Dominant model (AA+GA vs. GG) model (d), Recesive model (AA vs. GA+GG) (e), Overdominant model (GA vs. GG+AA) (f), and Allelic model (A vs. G) (g).

(a)

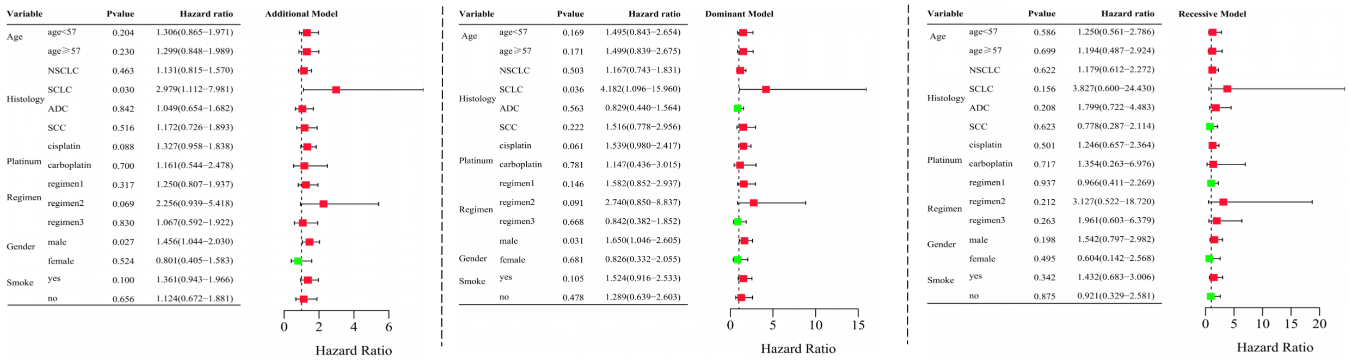

(b)

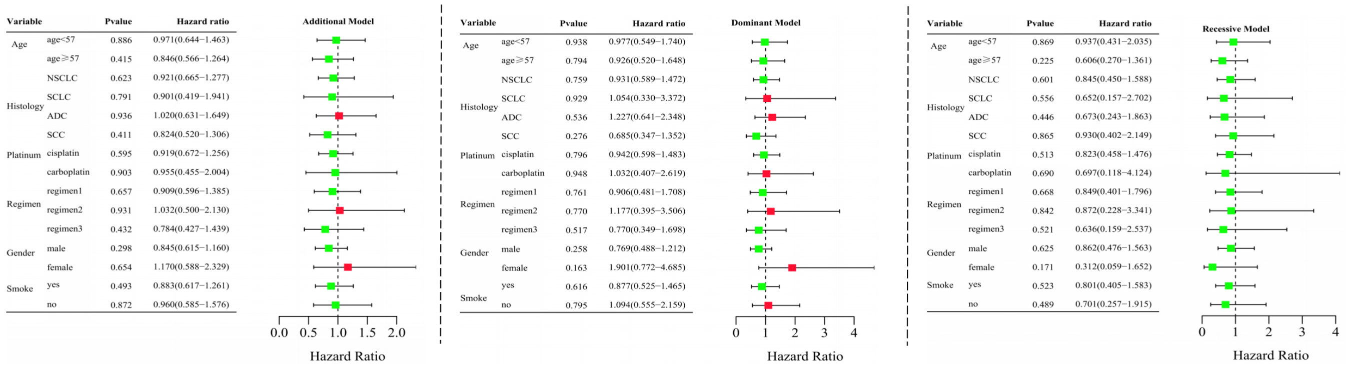

Supplemental Figure S4. Stratification analyses were conducted to examine the relationship between RETN polymorphisms rs1862513 (a) and rs3745367 (b) and overall toxicity in lung cancer patients undergoing platinum-based chemotherapy. The analyses were performed using additive, dominant, and recessive genetic models, while adjusting for various confounding factors including age, sex, disease stage, histological type, smoking status, and specific chemotherapy regimens. The results are depicted with each box and horizontal line representing the odds ratio (OR) and its 95% confidence interval (CI), respectively. The study focused on different types of lung cancer, such as non-small cell lung carcinoma (NSCLC), adenocarcinoma (ADC), squamous cell carcinoma (SCC), and small cell lung cancer (SCLC). The chemotherapy regimens considered were: Regimen 1, which combines platinum with gemcitabine; Regimen 2, platinum with etoposide; and Regimen 3, platinum with pemetrexed.

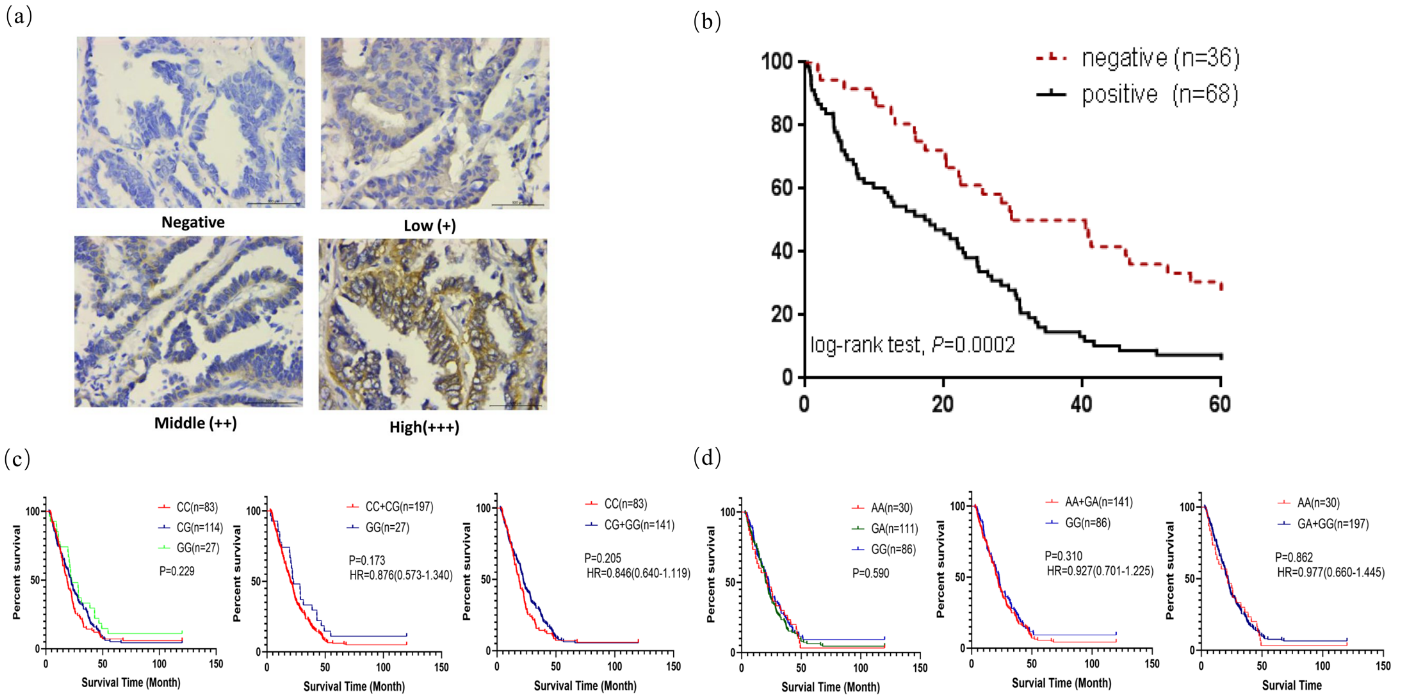

Supplemental Figure S5. Correlation between resistin expression and polymorphism and 5-year overall survival. (a) Representative immunohistochemical staining of resistin expression in human lung adenocarcinoma tissues; (b) Overall survival analyses for lung adenocarcinoma patients based on the expression of resistin in adenocarcinoma tissues according to Kaplan-Meier's method; Genotype of *RETN* rs1862513 (c) and rs3745367(d) and their association with overall survival.

Supplemental Table S1. Clinical characteristics of lung cancer patients and healthy controls

Characteristics	Patients, n(%) (n=498)	Controls, n(%) (n=213)	*P*
Gender			
Male	394(79.1)	80(37.6)	0.000*
Female	104(20.9)	133(62.4)	
Age (years)			
< 57	242(48.6)	74(34.7)	0.000*
≥ 57	256(51.4)	139(65.3	
Histology			
NSCLC	429(86.1)		
SCLC	69(13.9)		
Other[a]	23(4.6)		
NSCLC			
SCC	189(37.9)		
ADC	217(43.6)		
Stage(NSCLC)			
I, II	13(3.0)		
III, IV	416(97.0)		

Stage(SCLC)	
Limited	36(52.2)
Extensive	33(47.8)
Regimen	
Regimen1	192(41.4)
Regimen2	68(14.6)
Regimen3	137(29.3)
Regimen4	27(5.8)
Regimen5	29(6.2)
Other[b]	14(3.0)
Chemotherapy Response	
Responder	184(39.4)
Non-responder	283(60.6)
Overall Toxicity	
Grade 0-2	286(61.2)
Grade 3-4	181(38.8)
Gastrointestinal toxicity	
Grade 0-2	366(78.4)
Grade 3-4	101(21.6)
Hematological Toxicity	
Grade 0-2	353(75.6)
Grade 3-4	114(24.4)

Abbreviations: n, number; SCC, squamous cell carcinoma; ADC, adenocarcinoma; SCLC, small cell lung cancer; Othera, mixed-cell or undifferentiated carcinoma; NSCLC, non-small cell lung cancer; Regimen1,platinum + gemcitabine; Regimen2, platinum + etoposide; Regimen3, platinum + pemetrexed; Regimen4, platinum + paclitaxel; Regimen5, platinum + docetaxel; Otherb, platinum + irinotecan or platinum + navelbine; *P < 0.05.

Supplemental Table S2. Characteristics of the included studies on *RETN* polymorphisms and cancer susceptibility

First Author	Year	Country	Ethnicity	Cancer type	Source of control	Genotyping method	NOS score	Number of cases	Number of control	Genotype of cases			Genotype of controls		
rs1862513										CC	CG	GG	CC	CG	GG
Alharithy	2014	Saudi Arabia	Asian	Colon cancer	HB	PCR-RFLP	7	60	60	15	33	12	24	20	16
Duzkoylu	2015	Turkey	Asian	Colorectal cancer	HB	PCR-RFLP	7	123	79	53	61	9	31	36	12
Gong	2023	China	Asian	Lung cancer	HB	MassARRAY	6	487	209	188	243	56	78	98	33
Hu	2017	China	Asian	Lung cancer	HB	Real-time PCR	6	371	451	164	157	50	182	203	66
Kohan	2017	Iran	Asian	Breast cancer	HB	PCR-RFLP	7	150	150	50	63	37	63	63	24
Mahmoudi	2014	Iran	Asian	Colorectal cancer	HB	PCR-RFLP	8	197	213	38	83	76	56	85	76

Mihajlovic	2019	Serbia	Caucasian	Colorectal cancer	HB	TaqMan	6	86	75	47	30	9	43	26	6
Muñoz-Palomeque	2018	Mexico	Caucasian	Breast cancer	HB	PCR-RFLP	8	100	308	53	42	5	199	102	7
Ozgor	2019	Turkey	Asian	Endometrial cancer	HB	PCR-RFLP	7	94	94	39	45	10	60	39	5
Pechlivanis	2009	Czech Republic	Caucasian	Colorectal cancer	HB	TaqMan	7	702	752	317	262	63	393	265	56
W˚ags¨ater	2008	Sweden	Caucasian	Colorectal cancer	PB	Real-time PCR	7	248	256	127	95	26	137	103	16
Wang	2020	China	Asian	Breast cancer	PB	TaqMan	7	515	541	214	205	96	224	241	76
Yang	2018	China	Asian	Oral squamous cell carcinoma	PB	TaqMan	8	935	1200	352	452	131	443	575	182
rs3745367										GG	GA	AA	GG	GA	AA
Alharithy	2014	Saudi Arabia	Asian	Colon cancer	HB	PCR-RFLP	7	60	60	3	51	6	15	39	6
Gong	2023	China	Asian	Lung cancer	HB	MassARRAY	6	490	208	180	239	71	85	86	37
Hu	2017	Taiwan	Asian	Lung cancer	HB	Real-time PCR	6	371	451	164	164	43	190	194	67
Mahmoudi	2016	Iran	Asian	Colorectal cancer	HB	Real-time PCR	7	172	192	35	72	65	78	88	26
Wang	2020	China	Asian	Breast cancer	PB	TaqMan	7	515	541	184	259	72	212	252	77
Yang	2018	China	Asian	Oral squamous cell carcinoma	PB	TaqMan	8	935	1200	349	449	137	461	561	178

Supplemental Table S3. Clinical Character of lung cancer patients with platinum-based chemotherapy

Clinical Characteristics	Number	Response			Total toxicity			Gastrointestinal toxicity			Hematological toxicity		
		Responders	Non-responders	P value	Grade 0-2	Grade 3-4	P value	Grade 0-2	Grade 3-4	P value	Grade 0-2	Grade 3-4	P value
All	467	283(60.6)	184(39.4)		286(61.2)	181(38.8)		366(78.4)	101(21.6)		353(75.6)	114(24.4)	
Gender				0.021*			0.512			0.031*			0.776
Male	371	215(75.97)	156(84.78)		230(80.4)	141(77.9)		299(81.7)	72(71.3)		282(79.9)	89(78.1)	
Female	96	68(24.03)	28(15.22)		56(19.6)	40(22.1)		67(18.3)	29(28.7)		71(20.1)	25(21.9)	
Age(years)				0.545			0.661			0.002*			0.572
<57	234	145(51.24)	89(48.37)		141(49.3)	93(51.4)		169(46.2)	65(64.4)		173(49.0)	60(52.6)	
≥57	233	138(48.76)	95(51.63)		145(50.7)	88(48.6)		197(53.8)	36(35.6)		180(51.0)	54(47.4)	
History of Smoking				0.045*			0.539			0.439			0.552
Yes	286	163(57.60)	123(66.85)		172(60.1)	114(63.0)		228(62.3)	58(57.4)		213(60.3)	73(64.0)	
No	181	120(42.40)	61(33.15)		114(39.9)	67(37.0)		138(37.7)	43(46.5)		140(39.7)	41(36.0)	

Histology				0.000*			0.067			0.069			0.535
NSCLC	371	241(85.16)	130(70.65)		237(82.9)	134(74.0)		299(81.7)	72(71.3)		284(80.4)	87(76.3)	
SCLC	68	26(9.19)	42(22.83)		34(11.9)	34(18.8)		48(13.1)	20(19.8)		50(14.2)	18(15.8)	
Other[a]	28	16(5.65)	12(6.52)		15(5.2)	13(7.2)		19(5.2)	9(8.9)		19(5.4)	9(7.9)	
NSCLC				0.000*			0.011*			0.774			0.005*
SCC	167	91(32.2)	76(41.3)		95(36.3)	72(39.8)		133(36.3)	34(33.7)		116(32.9)	51(44.7)	
ADC	204	150(53.0)	54(29.3)		142(45.4)	62(34.2)		166(45.4)	38(37.6)		168(47.6)	36(31.6)	
Stage (NSCLC)				0.868			0.725			0.629			0.155
Early Stage (I, II)	12	8(2.8)	4(2.2)		7(2.4)	5(2.8)		9(2.5)	3(3.0)		7(2.0)	5(4.4)	
Advanced Stage (III, IV)	387	249(88.0)	138(75.0)		245(85.7)	142(78.5)		312(85.2)	75(74.2)		295(83.6)	92(80.7)	
Stage (SCLC)				0.234			0.467			0.145			0.889
Limited Stage	35	11(3.9)	24(13.0)		19(6.6)	16(8.8)		26(7.1)	9(8.9)		26(7.4)	9(7.9)	
Extensive Stage	33	15(5.3)	18(9.8)		15(5.2)	18(9.9)		19(5.2)	14(13.9)		25(7.1)	8(7.0)	
Chemotherapy regimen				0.000*			0.001*			0.412			0.001*
Regimin1	192	110(38.87)	82(44.57)		105(36.7)	87(48.1)		149(40.7)	43(42.6)		131(37.1)	61(53.5)	
Regimin2	68	25(8.83)	43(23.37)		32(11.2)	36(19.9)		50(13.7)	18(17.8)		48(13.6)	20(17.5)	
Regimin3	137	102(36.04)	35(19.02)		100(35.0)	37(20.4)		110(30.1)	27(26.7)		117(33.1)	20(17.5)	
Regimin4	27	18(6.36)	9(4.89)		21(7.3)	6(3.3)		24(6.5)	3(3.0)		23(6.5)	4(3.5)	
Regimin5	29	20(7.07)	9(4.89)		21(7.3)	8(4.4)		24(6.5)	5(4.9)		26(7.4)	3(2.6)	
Other[b]	14	8(2.83)	6(3.26)		7(2.4)	7(3.9)		9(2.5)	5(4.9)		8(2.3)	6(5.3)	

Abbreviations: NSCLC = non-small-cell lung cancer; SCC= squamous-cell carcinoma; ADC = adenocarcinoma; SCLC = small-cell lung cancer; Other[a] = mixed-cell or undifferentiated carcinoma; Regimin1 = platinum + gemcitabine; Regimin2 = platinum + etoposide; Regimin3 = platinum + pemetrexed; Regimin4 = platinum + paclitaxel; Regimin5 = platinum + docetaxel; Other[b] = platinum + irinotecan or platinum + navelbine; N = number;

*P < 0.05
